# Adapting to the Changing Climate: An Assessment of Local Health Department Preparations for Climate Change-Related Health Threats, 2008-2012

**DOI:** 10.1371/journal.pone.0151558

**Published:** 2016-03-18

**Authors:** Connie Roser-Renouf, Edward W. Maibach, Jennifer Li

**Affiliations:** 1 Center for Climate Change Communication, George Mason University, Fairfax, Virginia, United States of America; 2 National Association of County and City Health Officials, Washington, District of Columbia, United States of America; Rutgers University, UNITED STATES

## Abstract

**Background:**

Climate change poses a major public health threat. A survey of U.S. local health department directors in 2008 found widespread recognition of the threat, but limited adaptive capacity, due to perceived lack of expertise and other resources.

**Methods:**

We assessed changes between 2008 and 2012 in local public health departments' preparedness for the public health threats of climate change, in light of increasing national polarization on the issue, and widespread funding cutbacks for public health. A geographically representative online survey of directors of local public health departments was conducted in 2011–2012 (N = 174; response rate = 50%), and compared to the 2008 telephone survey results (N = 133; response rate = 61%).

**Results:**

Significant polarization had occurred: more respondents in 2012 were certain that the threat of local climate change impacts does/does not exist, and fewer were unsure. Roughly 10% said it is not a threat, compared to 1% in 2008. Adaptation capacity decreased in several areas: perceived departmental expertise in climate change risk assessment; departmental prioritization of adaptation; and the number of adaptation-related programs and services departments provided. In 2008, directors' perceptions of local impacts predicted the number of adaptation-related programs and services their departments offered, but in 2012, funding predicted programming and directors' impact perceptions did not. This suggests that budgets were constraining directors' ability to respond to local climate change-related health threats. Results also suggest that departmental expertise may mitigate funding constraints. Strategies for overcoming these obstacles to local public health departments' preparations for climate change are discussed.

## Background

"Climate change, once considered an issue for a distant future, has moved firmly into the present" ([1], p. 1). So begins the National Climate Assessment, warning Americans of the immediacy of a threat many in the U.S. still regard as remote [2].

Global climate change is one of the most serious public health threats facing the United States [1, 3, 4] and the global community [5, 6], endangering human health in multiple ways, including increases in the frequency and intensity of extreme weather events, increased transmission and geographic expansion of vector-borne diseases, and compromised air and water quality [1, 7]. U.S. Physicians already report increases among their patients in climate change-related allergic symptoms, chronic lung disease severity, and injuries from extreme weather and heat [8, 9, 10]; these impacts are expected to increase over the coming century [1]. Because local physical and social conditions can moderate or exacerbate the health impacts of climate change on human populations, local public health department (LHD) preparedness–in terms of expertise, services and programming–is central to adaptation to the changes that are occurring.

As an "amplifier of existing health risks," rather than a stand-alone risk factor [6], climate change may create significant public health needs that exceed local capacity to respond, heightening the demands placed on programs and services local health departments have long provided [11]. Programs addressing, for example, heat-related illnesses, vector- and water-borne diseases, and housing needs for people displaced by extreme weather events may be more heavily burdened, and new programs may be needed.

Most public health professionals are aware of the threat, but the impacts that specific communities will face are still imperfectly understood [11, 12], and few local health departments have made climate change adaptation a top priority, believing it to be a less pressing threat than other health hazards [13, 14, 15]. Thus, many health departments remain in the early stages of adapting to the changing climate. As local impacts become clearer and more severe over time, departmental focus on developing response strategies is likely to increase, but proactive preparations can reduce the harm from particular climate impacts, such as extreme weather [16].

Because the health impacts of climate change are experienced locally, the involvement of local communities in monitoring, discussing, advocating, and assisting with climate change adaptation is crucial. To assess city and county health department readiness to address local climate change impacts, a sample of local health department directors were surveyed in 2012 regarding climate change impacts within their jurisdictions, their departments' prioritization of preparing for climate change, and their departments' adaptation-related programming and expertise. The progress LHDs are making in preparing for climate change impacts was assessed by comparing the 2012 results to a similar sample surveyed in 2008, examining differences in climate change beliefs, perceived expertise and programmatic activities, and focusing on two specific obstacles to LHD adaptation efforts: limited resources and low risk perceptions.

The National Climate Assessment outlines a number of obstacles to adaptation: Climate change projections can be difficult to use in local decision-making; the resources needed to begin and maintain adaptation efforts may be lacking; coordination and collaboration within and across political systems and natural boundaries may be difficult; and institutional constraints and a lack of leadership may hinder efforts, as may divergent risk perceptions and values [17].

While all of these obstacles may have slowed adaptation over the four years between the two surveys, two stand out: The nation experienced a major recession leading to major funding cuts for many public health programs and social services, thereby reducing the available resources for adaptation; and increasing polarization on the issue of climate change led to decreases in the proportion of the U.S. public recognizing the reality, human causes and potential harm of climate change. The survey results suggest that these created barriers that have hindered progress toward climate change adaptation within LHDs.

Below we briefly review the 2008 survey results, the changes between 2008 and 2012 in funding for public health that occurred in response to the recession, and the polarization of public opinion on climate change in the U.S. that occurred during the same time period. Both of these events can help to explain shifts in LHD directors' attitudes, and to the number of programs and services their departments offer.

### The 2008 Local Health Department Director Survey: *Are We Ready*?

In 2007–2008, to assess local health departments' readiness to meet the challenges of climate change, the Environmental Defense Fund, the National Association of County and City and County Health Officials (NACCHO) and George Mason University surveyed a geographically representative sample of local health department directors. The study–*Are We Ready*?*–*examined the perceptions of local health department directors regarding local effects of climate change, and their departments' readiness to respond [13, 15]. Results pointed to widespread awareness of the importance of climate change for public health, but a lack of activities to detect, prevent or ameliorate climate-related health problems. Most directors believed that climate change was already occurring locally, and would cause a serious local health problem within 20 years; few thought, however, that their departments had the expertise to address the threat.

These results have been replicated and extended in several other surveys, including surveys of the chief health officers of state and territorial health agencies [18], public health nursing administrators [19], California public health officers [20], Oregon public health officers [21], New York LHD officers [22], and environmental health directors at local, state and territorial public health agencies [23]. The results in all these surveys are similar, reporting limited preparedness for the health impacts of climate change: Half of the nursing administrators, for example, believed their departments have the responsibility to address climate-related health threats, but three-quarters said they were unprepared to do so [19]. Seventy percent of California public health officers said they do not have adequate information to respond to climate change, although 94 percent perceived it to be a health threat [20]. Less than 20 percent of the state/territorial health officers said their agencies had the capacity to assess, plan or respond to climate change [18]. And two-thirds of the environmental health directors said their departments had low preparedness for climate change [23]. The cumulative conclusion from these surveys is that public health professionals in the U.S. have felt ill–equipped to help their jurisdictions adapt to the changing climate.

### Funding Cuts

Although the need to expand expertise and programmatic activities relating to the health impacts of climate change was clear, the economic recession led to major funding cutbacks for public health departments [24]. More than 52,200 state and local public health jobs were lost in the U.S. following 2008; these losses represented 17 percent of the state and territorial health department workforce and 22 percent of the local health department work force [25]. In 2011 alone, 23 percent of local health departments reduced or eliminated clinical health services programs, 23 percent reduced or eliminated emergency preparedness services, 19 percent reduced or eliminated immunization programs, and 19 percent reduced or eliminated population-based primary prevention programs. Over half of local health departments reduced or eliminated at least one program (57%) [26]. In short, funding reductions made it unlikely that adaptation-related programs in local health departments would expand, or that expertise in climate change adaptation would increase.

### Issue Polarization and Waning Concern

Funding, however, was not the only barrier to improved climate change preparedness in the nation’s health departments. Concurrent with the funding cutbacks, the U.S. public's recognition of and concern about climate change dropped, and attitudinal polarization on the issue increased.

Multiple polls conducted between 2008 and 2010 documented declines in belief that climate change is occurring–an average decrease of 16 percentage points during this time span [27]. The declines were found primarily among conservatives; Gallup poll data, for example, show that in 2008, 50 percent of conservatives and 73 percent of liberals believed that the effects of global warming had already begun–a gap of 23 percentage points; by 2010, the gap had widened to 44 percentage points, with 30 percent of conservatives and 74 percent of liberals saying that effects had begun [28].

While we have no prior evidence that this polarization occurred within the public health community over this time period, a link between political ideology and climate change beliefs has been identified among public health professionals: Politically conservative public health nurses [19] and environmental health directors [23] have lower risk perceptions for climate change health impacts than those who are more liberal politically.

These results have implications for health department preparedness because perceived risks are strongly associated with protective behavior [29, 30, 31]; to the extent that risk perceptions are low, protective actions are less likely across a wide range of threats. Specific to public health, Syal and colleagues found that climate change risk perceptions explained 27 percent of the variance in the number of adaption-related programmatic activities within environmental health directors' departments, and that political ideology was strongly predictive of risk perceptions [23]. Thus, to the extent that climate change attitudes within the public health community reflect trends among the broader American public, attitudes may have polarized; and if so, risk perceptions and efforts to build programs to detect and protect local populations from climate change may have decreased among LHDs with administrators who doubt the reality and threat of climate change.

## Research Questions

In light of prior research indicating limited preparedness of LHDs to address the health effects of climate change, and the barriers that arose between 2008 and 2012 through funding cutbacks and polarization in public concern, we explored the following research questions:

1. RQ1: Between 2008 and 2012, how did preparedness for climate-related health threats in local health departments change in terms of:
  - RQ1a: the level of threat perceived;
  - RQ1b: expertise in climate-related risk assessment and program planning; and
  - RQ1c: adaptation-related programmatic activity?
2. RQ2: How strongly are perceptions of the local threats of climate change and departmental expertise related to adaptation-related programming and services within local public health departments, and did this relationship change between 2008 and 2012?
3. RQ3: How strongly are local health department budgets related to the adaption-related programming within their departments, and did this relationship change between 2008 and 2012?

## Methods

Of the factors shaping adaptation responses described within the National Climate Assessment [17], we focused on two: *resources*, as reflected in LHD departments' *budgets* and in LHD directors' perceptions of the *adaptation expertise* within their departments and state; and *risk perceptions*, as reflected by LHD directors' perceptions. While expertise and budget do not capture all the resources needed for adaptation, we feel they are the most likely to have constrained adaptation between 2008 and 2012.

The survey data were gathered through an online, geographically representative survey of local health department (LHD) directors, using a stratified random sample of 350 LHDs [32]. The sampling plan included 12 strata based on U.S. Census Region (Northeast, Midwest, South, West) and population of LHD jurisdiction (<50,000, 50,000 to 499,999, 500,000+). The Human Subjects Review Board at George Mason University reviewed and approved the study procedures and questionnaire prior to data gathering, including the recruitment and consent procedures.

The survey instrument was largely a replication of close-ended items drawn from the 2008 LHD director survey, *Are We Ready*. Questions assessed the directors' perceptions of the impacts of climate change within their jurisdictions; the priority of climate change adaption within their departments; and their assessments of their departments' and states' expertise for responding to the impacts of climate change. The primary dependent variable was the number of programs operated by the health departments that address the health threats that are expected to increase due to climate change, such as heat waves, flooding and vector-borne infectious disease; we refer to these as adaptation-related programs. For more detail on measurement, please see the Appendix.

The survey took an average of ten minutes to complete, and was pre-tested for length and clarity prior to fielding with the NACCHO Environmental Public Health Tracking Workgroup. Local public health directors selected for the sample first received a letter describing the survey. The letter stated that George Mason University and NACCHO were conducting a follow-up to the 2008 survey and requested their participation. The letter said that participation was entirely voluntary, the survey would be brief, and that their identities, while known to the first author, would be protected. The letter concluded with a statement that they would be receiving an email with a link to the survey within the next few days. The email repeated all the information in the letter, and supplied a link to the survey; clicking on the link was interpreted as the respondent's consent to participate.

The email addresses of the respondents gave the first author access to information on the respondent's jurisdiction location and size; once the surveys were matched to the jurisdiction information, the names and email addresses were deleted and were not shared with anyone.

A total of 174 LHD directors responded to the survey (response rate = 49.7%). Three people refused to participate, and 16 completed only the first few items on the survey and left all other items blanks, reducing the number of respondents for whom we have complete data to 158.

The 2008 *Are We Ready* survey–used for comparison in this research–had the same sampling frame and sampling strategy as the current survey (i.e., LHD directors); 250 directors were selected for participation in a telephone survey lasting an average of 45 minutes. The survey had a response rate of 61 percent. For more details on the initial survey's methods, see Maibach and colleagues, 2008 [13].

### Treatment of the Data

The 2008 and 2012 data were merged and analyzed in SPSS; analysis of variance and Levene's statistic were used to assess changes in means and standard deviations. Indices were created for the number of perceived local climate change impacts ($\bar{X}$ = 3.00 of 12 possible; *sd* = .71; α = .86); perceived expertise (six Likert-type items; $\bar{X}$ = 2.07 on 1–4 scales, where 4 indicates high self-assessed expertise; *sd* = .65; α = .90); and number of adaptation-related programs ($\bar{X}$ = 7.22 of 12 possible; *sd* = 2.57; α = .72). Health department budget was assessed on a 3-point scale (1 = Less than $1M; 2 = $1M to $4.99M; 3 = $5M or more; *$\bar{X}$ = 2.09; sd* = .84).

Regression analysis was used to examine the association of three independent variables–department budget, perceived departmental expertise, and number of perceived local climate change impacts–with two dependent variables in 2008 and 2012: priority of climate change within the directors' health department, and number of adaptation-related programs the department had in place. U.S. census region (Northeast, Midwest, Southern and Western) and type of jurisdiction (metropolitan, non-metropolitan and mixed) were coded as dummy variables and entered into the regression as controls; Western and mixed jurisdictions were the omitted categories in the regressions. The surveys were analyzed separately to assess changes in the importance of budget, perceived impacts and expertise over time. Examination of collinearity diagnostics indicated that collinearity was not an issue.

## Results

### Sample Characteristics

Table 1 shows the budgets, regions and types of jurisdiction for the 2008 and 2012 samples. The samples did not differ significantly in regional or metropolitan/rural distributions. The 2012 sample had significantly lower budgets (*2008 budget $\bar{X}$ = 2.28; sd = .79; 2012 budget $\bar{X}$ = 1.96; sd = .86; t = 3.28 p < .001),* but the variance did not change.

**Table 1:** Sample Characteristics.

	Percentages		
	2008	2012	Total
**Budget**			
Less than $1,000,000	20.8	38.8	31.2
$1,000,000 to $4,999,999	30.4	26.5	28.1
$5,000,000 or more	48.8	34.7	40.7
**Region**			
Northeast	23.7	23.5	23.6
Midwest	25.4	22.5	24.1
South	28.1	30.4	29.2
West	22.8	23.5	23.1
**Metro Jurisdiction**			
Non-metro	41.1	47.1	44.5
Mixed	4.7	3.6	4.1
Metro	54.2	49.3	51.4
*N*	*133*	*174*	*307*

### Local Impacts

Mean perceptions of local climate change impacts and beliefs about future impacts changed little between 2008 and 2012 (see Table 2). In 2008, 69 percent of the directors believed climate change was having local impacts, as compared to 66 percent in 2012. The proportion who believed their jurisdiction would experience climate change over the next two decades was 78 percent in 2008 and 76 percent in 2012. In 2008, 59 percent believed a serious climate-related health problem would occur in their jurisdiction over the next two decades, as compared to 61 percent in 2012.

**Table 2:** Perceptions and Expectations of Local Climate Change Impacts, 2008–2012.

	Percentages						
	Strongly agree	Somewhat agree	Somewhat disagree	Strongly disagree	Don't know	Mean^a^	SD
***My jurisdiction has experienced climate change in the past 20 years*.**							
2008	9.0	60.2	10.5	.8	19.5	*2*.*96*	*0*.*53*
2012	18.4	47.5	8.9	12.0	13.3	*2*.*83*	*0*.92***
*Change*	*+9*.*3*	*-12*.*7*	*-1*.*7*	*+11*.*3*	*-6*.*3*		
***My jurisdiction will experience climate change in the next 20 years*.**							
2008	22.6	55.6	2.3	.8	18.8	*3*.*23*	*0*.*54*
2012	39.2	37.3	6.3	9.5	7.6	*3*.*15*	*0*.94***
*Change*	*+16*.*7*	*-18*.*3*	*+4*.*1*	*+8*.*7*	*-11*.*2*		
***In the next 20 years*, *it is likely that my jurisdiction will experience one or more serious public health problems as a result of climate change*.**							
2008	11.3	48.1	8.3	1.5	30.8	*3*.*00*	*0*.*61*
2012	29.1	32.3	13.3	10.8	14.6	*2*.*93*	*1*.00***
*Change*	*+17*.*8*	*-15*.*8*	*+5*.*0*	*+9*.*3*	*-16*.*3*		

^a^Means are on a four-point scale;

1 = strongly disagree; 4 = strongly agree; "don't know" responses excluded from the analysis. Significance tests in mean column are for the difference in means between 2008 & 2012; significance tests in the SD column are for the differences in standard deviations between 2008 & 2012.

***p < .001

These proportions mask an important change: The distribution of responses changed significantly, with more directors responding at the ends of the scales–strongly agreeing or strongly disagreeing–and fewer choosing the middle scale points or saying they "don't know." The proportion who *strongly* agreed that their jurisdiction had experienced climate change doubled from nine to 18 percent, while the proportion who *strongly* disagreed jumped from one to 12 percent; "don't know" responses decreased from 19 to 13 percent. A similar pattern was found in expectations for future climate impacts and climate-related public health problems. The decrease in 'don't know' responses suggests attitude formation, while attitudinal polarization is seen in the increase in responses at the ends of the scales. Standard deviations on all three items were significantly higher in 2012 than 2008 (for perceived local climate change, *Levene statistic = 30*.*22;* for anticipated impacts *= 21*.*10;* and for anticipated public health problems *= 32*.*52;* all three items, *p <* .*001)*.

### Expertise

In 2008 many directors reported that they lacked expertise and resources to address the local public health impacts of climate change, and four years later, they reported even less. Their perceptions of their own knowledge about climate change did not change significantly from 2008 to 2012, with 66 percent in 2008 and 59 percent in 2012 believing they had sufficient expertise *(F = 2*.*25*, *n*.*s*.*)*. The proportion, however, who believed their colleagues' had adequate knowledge of local climate change impacts decreased; 46 percent felt their colleagues had sufficient expertise in 2008, as compared to 36 percent in 2012 *(F = 11*.*91*, *p <* .*001)*. The proportion saying that their departments had sufficient risk assessment expertise fell from 23 percent to 19 percent; *(F = 4*.*99*, *p <* .*05)* and agreement that their state health departments' ability could help them develop adaptation plans fell from 57 percent to 46 percent *(F = 3*.*79*, *p <* .*05;* Table 2). The variance of perceptions widened significantly on all expertise items, with the exception of perceived state health department expertise. (See Table 3.)

**Table 3:** Perceptions of Adaptation Expertise and Departmental Readiness.

	Percentages						
	Strongly agree	Somewhat agree	Somewhat disagree	Strongly disagree	Don't know	Mean^a^	SD
***I am knowledgeable about the potential public health impacts of climate change*.**							
2008	4.5	61.4	28.8	2.3	3.0	*2*.*70*	.*59*
2012	14.7	44.2	19.9	19.2	1.9	*2*.*56*	.*97* ***
*Change*	*+10*.*2*	*-17*.*1*	*-8*.*9*	*+17*.*0*	*-1*.*1*		
***The other relevant senior managers in my health department are knowledgeable about the potential public health impacts of climate change*.**							
2008	3.8	42.0	36.6	5.3	12.2	*2*.*50*	.*68*
2012	6.4	29.5	30.8	28.2	5.1	*2*.*15* ***	.93***
*Change*	*+2*.*6*	*-12*.*5*	*-5*.*9*	*+22*.*9*	*-7*.*1*		
***My health department currently has ample expertise to assess the potential public health impacts associated with climate change that could occur in my jurisdiction*.**							
2008	3.8	18.8	49.6	27.8	0	*1*.*98*	.*79*
2012	5.1	13.9	29.1	46.8	5.1	*1*.76*	.89***
*Change*	*+1*.*3*	*-4*.*9*	*-20*.*5*	*+19*.*0*	*+5*.*1*		
***My health department currently has ample expertise to create an effective plan to protect local residents from the health impacts of climate change*.**							
2008	15.9	51.5	31.8	0	.8	*1*.*86*	.*70*
2012	12.3	34.4	45.5	3.2	4.5	*1*.*75*	.85**
*Change*	*-3*.*6*	*-17*.*1*	*13*.*6*	*+3*.*2*	*+3*.*8*		
***My state health department currently has ample expertise to help us create an effective plan in this jurisdiction to protect residents from the health impacts of climate change*.**							
2008	22.6	34.6	18.8	21.1	3.0	*2*.*12*	.*82*
2012	16.1	29.7	34.8	14.2	5.2	*1*.90*	.*91*
*Change*	*-6*.*4*	*-4*.*9*	*16*.*0*	*-6*.*9*	*+2*.*2*		
***Preparing to deal with the public health effects of climate change is an important priority for my health department*.**							
2008	12.0	39.1	40.6	3.8	4.5	*2*.*62*	.*75*
2012	16.5	24.7	24.7	28.5	5.7	*2*.*31* **	*1*.*08* ***
*Change*	*+4*.*4*	*-14*.*4*	*-15*.*9*	*+24*.*7*	*+1*.*2*		

^a^ Means are on a four-point scale;

1 = strongly disagree; 4 = strongly agree; "don't know" responses excluded from the analysis. Significance tests in mean column are for the difference in means between 2008 & 2012; significance tests in the SD column are for the differences in standard deviations between 2008 & 2012.

*p < .*05;*

**p < .*01;*

***p < .*001*

### Adaptation Priority

The departmental priority of preparing for climate change impacts declined significantly between 2008 and 2012 from 2.62 to 2.31, below the mid-point of the 4-point scale *(F = 7*.*51*, *p <* .*01;* Table 3). The proportion who strongly disagreed that preparing for climate change impacts was an important priority within their health department jumped by 25 percentage points, from 4 percent to 28 percent. The variance increased significantly as well, indicating a greater diversity of prioritization.

### Adaptation-Related Programmatic Activities

The number of local impacts perceived by the directors did not change significantly from 2008 to 2012 *(2008 $\bar{X}$ = 3.90; 2012 $\bar{X}$ = 3.82; 12 possible; F = .03, n.s.),* but the number of adaptation-related programmatic activities decreased significantly, from an average of 7.75 to 6.74 of the 12 services assessed *(F = 11*.*06*, *p <* .*001);* a marginally significant change in the standard deviations suggests that some departments saw greater declines than others (*Levene statistic = 2*.*91*, *p <* .*10)*.

### Predictors of Local Health Department Responses to Climate Change

The regression results shown in Table 4 paint a picture of changing conditions over time: perceived local impacts of climate change decreased in importance between 2008 and 2012 as a predictor of adaption-related programmatic activities, while departmental budget became a more powerful predictor.

**Table 4:** Predictors of Climate Change Priority, and Adaptation-Related Programs and Services.

	Climate Change Departmental Priority^a^		Adaptation-Related Programs^a^	
	2008	2012	2008	2012
North-East Region	.02	-.13	.07	-.20**
Mid-West Region	.19	-.02	.10	-.05
Southern Region	-.01	-.10	-.05	-.07
Non-Metropolitan Jurisdiction	.00	-.08	.08	.20*
Metropolitan Jurisdiction	-.18	-.13	-.02	.00
Department Budget	.06	-.08	.03	.22**
Number of Perceived Local Impacts	.28**	.42***	.30**	.14
Perceived Adaptation Expertise	.32***	.40***	.19*	.29***
*Adjusted R*^*2*^	.*220*	.*309*	.*178*	.*204*
*F*	*5*.05***	*8*.71***	*2*.89**	*5*.51***
*N*	*(115)*	*(139)*	*(116)*	*(142)*

^a^Cell entries are standardized regression coefficients.

*p < .05;

**p < .01;

***p < .001.

At both time points, the priority accorded to preparing for climate change was strongly associated with both the number of perceived impacts and the directors' assessments of their departments' adaptation expertise: In health departments where directors believed that climate change was having more impacts, and that they, their colleagues, and their state health department had the expertise to protect the jurisdiction, preparing for the health impacts of climate change was a higher priority, explaining 22 percent of the variance in 2008 and 40 percent in 2012. The increase in the adjusted R^2^ may be interpreted in part as an artifact of the larger variance in priority in 2012, but it also suggests that as the priority of adaptation fell in many local health departments, adaptation priority remained high in departments where the directors believe climate change impacts are higher and where response expertise was perceived to be high. These results held across census regions and types of jurisdictions, as none of these variables were significantly related to climate change priority.

The health department's budget was unrelated to adaptation priority at either time point. Budget did, however, play an important part in predicting the number of adaptation-related programs and services of local health departments in 2012: Programming was unrelated to departmental budget in 2008 *(β =* .*03*, *n*.*s*.), while perceived local impacts had a strong relationship to programming *(β =* .*30*, *p <* .*01)*. By 2012, the situation was reversed: a health department's budget more strongly predicted adaptation-related programs *(β =* .*22*, *p <* .*01)* than did the number of perceived local impacts of climate change *(β =* .*14*, *n*.*s*.*)*. This result suggests that directors' perceptions of local threats became less important over time in shaping their departments' adaptation-related programming; instead, funding was constraining the programs and services that health departments offered–a constraint reflected in the reduced number of adaptation-related programs and services local health departments provided.

Expertise, however, grew in importance as a predictor of adaptation-related programming, and was the strongest predictor of adaptation-related programming in 2012 *(β =* .*29*, *p <* .*001)*. More adaptation-related programs and services were in place in health departments where the staff had the expertise to assess the threat of climate-related impacts and their state health department could provide support; this is a stronger predictor than budget.

Between 2008 and 2012, regional and jurisdictional differences in the number of adaptation-related programs and services emerge that were previously absent: LHDs in Northeastern states had significantly fewer programs and services than other states *(β = -*.*20*, *p <* .*01);* and non-metropolitan jurisdictions had more programs and services than metropolitan and mixed jurisdictions *(β =* .*20*, *p <* .*05)*. These results suggest that greater programmatic losses were experienced in Northeastern LHDs, while fewer occurred in non-metropolitan jurisdictions.

Overall, the results suggest that in the face of budget cuts, directors who perceived multiple local threats from climate change lost some ability to preserve the programs that will help their jurisdictions adapt; those in departments and states with higher adaptation expertise, however, were better able to maintain their programs.

## Discussion

### Risk Perceptions, Polarization and Preparedness

The analysis shows that between 2008 and 2012, the growing attitudinal polarization on climate change in the U.S. [28] extended into the public health community: The proportions of LHD directors stating with certainty that their jurisdictions were/were not vulnerable to the public health threats of climate change increased, while the proportion who were less certain decreased. Although we assessed the polarization of risk perceptions with two cross-sectional datasets (as opposed to two surveys of the same respondents at the two different time points), the fact that our findings are consistent with multiple national surveys showing polarization during this time period lends strength to the inference.

Our results suggest that roughly ten percent of LHD directors doubted the threats posed by climate change to their communities. Directors who perceived fewer local impacts reported having fewer programs in place to address the threats, a finding consistent with prior research showing that environmental health directors with lower climate change risk perceptions had fewer adaptation-related programs in their jurisdictions [23].

LHD directors are community leaders responsible for protecting the health of the populations in their jurisdictions, and as Frumkin, Hess and Luber note [33], the first step in addressing a public health threat is recognition that it exists. Hence, educating the public health community about the reality of climate change's health effects should be a priority for professional enhancement and continuing education programs in public health.

Changing people's beliefs about climate change is, in general, challenging because it is rooted in deeply held values [34], and responsive to cues from political elites who may not accept the scientific evidence on climate change or the need for a national response [35]. Recent research has shown, however, that messages explaining the scientific consensus on climate change are effective in changing key climate change beliefs and fostering support for a response [36]. Public health professionals should be particularly receptive to consensus messages as they have been trained to implement policies grounded in scientific evidence; moreover, the precautionary principle that underlies public health practice instructs them to act–even in the absence of scientific consensus–if evidence of harm exists [37], which it demonstrably does [1, 5]. Given the high level of agreement among climate scientists on the reality, causes and threat of climate change, a failure to respond to its threat is contrary to the principles that guide public health practice.

Scientific consensus messages from the relevant national health agencies to local health departments may counter-balance statements from political elites who do not recognize the threat of climate change, as these agencies are likely to be viewed as highly credible and trustworthy by public health professionals. Evidence of local impacts and projections for future local impacts can also be effective in increasing directors' concern [38], as well as helping them to plan for the particular health threats their communities face.

### Preparedness and Funding

While failure to acknowledge and prepare for climate change impacts is a serious issue, recognizing the danger but lacking the resources to respond is a much more widespread problem. Between 2008 and 2012, there was a marked decline in LHD directors' self-assessed readiness to adapt to the changing climate, including declines in perceived adaptation expertise, priority, programming and the number of adaptation-related services health departments offered. Moreover, the decreases in programming and services reported here likely underestimate the actual decline in preparedness, as they only account for programs that were entirely eliminated–not those that have been reduced.

The declines in reported expertise are likely to be attributable to losses in health department staff, given the massive cutbacks to public health funding that occurred during the recession. Although we lack data on funding cutbacks within our respondents' departments to assess whether this is the case, it is apparent from the regression results that funding is now related to the number of adaptation-related programs and services offered by LHDs, in contrast to 2008, when it was unrelated.

Funding cuts have slowed recently, but have not been reversed. In 2013 more than a quarter (27%) of local health departments nationwide reported that their fiscal-year budgets had decreased from the prior year; a similar proportion (28%) anticipated budget cuts in 2014. NACCHO estimates that from 2008 through 2013, LHDs lost 48,300 jobs by layoffs and attrition [24]. Thus, although the National Climate Assessment [1] warns that climate change impacts are now occurring in the U.S., the public health agencies that are tasked with protecting communities from these impacts are constrained by a lack of resources that would enable them to expand adaptation-related programming and services.

### Moving Forward

The budgetary and perceptual barriers to adaptation that we have explored in this analysis may be interpreted in light of a recent review of the constraints and barriers to climate change adaptation [14]: Huang and colleagues identified multiple obstacles to increasing preparedness for climate-related health impacts, including uncertainty about the specific types and time-frames for impacts; inadequate funding; unequal access to technologies, such as GIS, that can assist in risk assessments and resource allocations; a lack of knowledge concerning the costs and effectiveness of various adaptation strategies; and low awareness of the risks. Most of these barriers could be addressed by increases in funding. Ebi and colleagues [39] recommended that federal funding for public health research in the area be expanded to more than $200 million annually to "understand, avoid, prepare for, and respond to the human health impacts of climate change in the United States" (p. 857), and concluded that current funding was inadequate to address the threat.

Climate change confronts public health departments with an array of new and increasing threats in a context constrained by limited funding, limited expertise, and uncertainty about the likelihood and magnitude of specific threats. Yet the public health discipline has a "toolbox" of well-honed methods suited to addressing these threats [33], developed as the field addressed other emergent threats. Multiple strategies–all of which have historical precedents within public health practice–are needed and may be called on to build community resilience. These include:

- **Risk assessment** to identify new and emerging health threats, such as increases in dengue fever, heat-related morbidity, drought and wildfires;
- **Development of response strategies,** including **policies** that contain and reduce local threats, such as community planning and zoning that increase resilience to extreme weather and sea level rise; and **treatments** for victims, such as medication and treatment plans for people infected with spreading vector-borne illnesses like Lyme Disease;
- **Public education campaigns** to reduce population exposure to local threats, such as the protective behaviors that may be taken during extreme weather events and intense heat waves.

The fact that mortality during heat waves has been higher in the Northeast and Midwest (which have more limited experience with extreme heat) than in the Southwest (where heat is a near-constant) [40], demonstrates that community resilience to at least some climate threats can be built. This will, however, take public health outside arenas it has traditionally occupied into, for example, energy, agriculture and community zoning [33]. Frumkin and colleagues suggest, for example, that public health practitioners should be collaborating with the transportation and agriculture professionals who are developing adaptation policies by providing them with information on the health implications of climate change [33]. In short, the combination of new and increasing threats mandates both established and innovative approaches.

A roadmap for the public health response to climate change has been developed by the U.S. Centers for Disease Control and Prevention: Building Resilience Against Climate Effects (the BRACE framework) [41]. The LHD directors' perceptions, when compared to the following steps of the framework, point to the strengths and weaknesses of local health department as they prepare for climate change:

- **Anticipate climate impacts and assess vulnerabilities:** Modeling of local impacts is improving, but there is still uncertainty about specific impacts within communities [1]; and although most LHD directors are aware of their jurisdictions' vulnerability, approximately a tenth do not recognize any local impacts. Ongoing climate research to improve our understanding of impacts at the community level, and continuing education for public health professionals can facilitate risk assessments by public health departments.
- **Project disease burden:** Quantitative modeling of increases in disease incidence requires specialized technology to which access is currently uneven, and many public health departments lack the skill sets needed to implement their use.
- **Assess public health interventions:** Reviewing the literature on prior interventions with related health threats requires staff who are both knowledgeable and available to conduct the research. Lack of information on effective adaptation strategies is currently a significant barrier to building resilience, which national health agencies could address by collecting and disseminating evaluations of interventions on climate-related health threats.
- **Develop and implement a climate action plan:** As with the assessment of prior interventions, this step entails staff with expertise; given the public health "toolbox," most public health professionals understand how to develop and implement action plans, though the threats they are addressing may be new to them and some of the effective strategies for addressing these threats may be unfamiliar.
- **Evaluate impacts to facilitate continuous quality improvement:** Evaluation of interventions is less likely when budgets are tight; without evaluations, however, our understanding of effective adaptation strategies will grow more slowly and locally generated knowledge will not be shared across communities. Funding for program evaluation should, therefore, be a component of community grants intended to build resilience to climate impacts.

In Wisconsin, the City of Milwaukee Health Department has responded to extreme heat events by working with the state's BRACE staff to develop the Excessive Heat Event Coordination Plan. The BRACE staff conducted a geo-spatial analysis of heat-related vulnerability in the greater Milwaukee urban area, using existing population and census data, natural and built environment data, and health factors to create a heat vulnerability index (HVI) that identifies the areas of greatest risk for negative health impacts due to extreme heat.

The HVI allows public health authorities in Milwaukee to direct prevention and intervention strategies, including risk messaging targeted to high risk populations, siting of cooling centers, and deployment of other resources. Additionally, as public health heat-related illness surveillance is critical, the City of Milwaukee Health Department can also request and analyze heat-related illness public health surveillance from area hospital emergency departments [42].

### Limitations and Future Research

The methods of data collection for the two LHD director surveys differed, which may have influenced the responses. In 2008, directors spoke on the phone to trained interviewers for an average of 45 minutes, answering many questions not reported here. In contrast, the 2012 survey took 10 minutes to complete and was completed by the director online. We also have limited information about the respondents that would permit a more thorough assessment of the comparability of the two samples; differences in the sizes of their jurisdictions, for example, is not known. Other obstacles to climate change adaptation efforts discussed by the National Climate Assessment, including institutional barriers and a lack of coordination and collaboration across and within organizations, may have contributed to the lack of progress we found, but were not assessed in the survey and are not accounted for in the data.

All self-report survey data may be colored by factors not assessed by the researchers or by conscious or unconscious biases in the survey respondents' answers. Our assessment of budget, in particular, was crude; information on the specific funding cutbacks experienced within health departments would lend strength to our finding that funding has become an increasingly important constraint on adaptation efforts. Higher risk perceptions for local impacts among the LHD directors might have mediated the constraints of limited resources, such that directors who perceived local health impacts would have retained more adaptation-related programs and services. We didn't find this, however, and it may be that low media coverage of climate change during the years between our two surveys [43] contributed to reducing directors' sense that climate change is an immediate threat, leading them to lower its departmental priority and sacrifice adaptation-related programs when cutbacks of some form were mandated. As the nation has recovered from the recession, the relationships among risk perceptions, funding and programmatic services may be again changing–a possibility to be hoped for and explored in future research.

## Conclusion

Bo Lim, a technical advisor on climate change adaptation with the United Nations Development Programme, has stated: "Adaptation is no longer tomorrow's choice, but today's imperative" [14]. Local health departments face this imperative if they are to protect vulnerable populations from the increasing heat, severe storms, droughts and flooding that climate change is bringing to their communities. As Frumkin and colleagues note, "responding to climate change will become an increasingly central part of public health" ([32], p. 251); and while public health agencies at the federal, state and local level must all respond to climate change, the key to preparedness and resilience lies in activities at the local level, as the health threats of climate change vary geographically. Without increases in funding to build expertise in risk assessment, and in program design and evaluation, however, adaptation may continue to stall.

## Appendix

### Measures

#### Department budget

Respondents were asked the approximate annual budget for their departments with three response categories: (1) Less than $1M; (2) $1M to $4.99M; or (3) $5M or more. Using NACCHO's 2010 data on the budgets of public health departments (NACCHO 2011) these categories were created to roughly trichotomize LHDs into equal groups. The 2012 respondents were somewhat more equally balanced on this measure (1 = 38%; 2 = 26%; 3 = 34%) than the 2008 respondents (1 = 20%; 2 = 29%; 3 = 46%). The skew in the 2008 data toward larger budgets likely reflects the cutbacks in funding that occurred after 2008.

#### Beliefs about Local Climate Change Impacts

Three Likert-type items assessed the degree to which respondents believed their jurisdiction had experienced climate change in the past 20 years; would experience it in the next 20 years; and would likely experience one or more serious local health threats in the next 20 years due to climate change. Four-point scales from "strongly disagree" (1) to "strongly agree" (4) were used, and a "don't know" option was also provided. The same scales were used for adaptation priority and perceived expertise.

#### Adaptation priority

One item assessed the priority of climate change adaptation in the health director's department.

#### Expertise

Six Likert-type items (with four-point response scales from "strongly disagree" (1) to "strongly agree (4) with a "don't know" option) were used to assess health directors' perceptions the expertise and resources available for assessing the risk climate change poses locally, and for developing an effective plan to protect their community. The mean of the six items, omitting the "don't know" responses, was used as an index of adaptation expertise (Cronbach's α = .90).

#### Perceived local impacts

Respondents shown a list of 12 public health issues and asked whether they believed climate change had affected each of the 12; responses indicating that climate change had increased the threat were summed to create an index of the number of perceived local impacts (Cronbach's α = .86). Note that *perceived* impacts are not the same as *actual* impacts, given the influence of prior beliefs on perceptions.

#### Adaptation-related programs

Twelve programmatic activities related to climate change health impacts were assessed. The question stem stated: "Below is a list of health issues that climate change may affect. For each of these health issues, please answer “Yes” if the health issue is currently an area of programmatic activity for your department. "Yes," "no," and "don't know" options were offered; "yes" items were summed to form an index (Cronbach's α = .72).

The dataset is available at the first author's page on Research Gate.
